# The Influence of Ultrasound on the Growth of *Nannochloris* sp. in Modified Growth Medium

**DOI:** 10.3390/life13020413

**Published:** 2023-02-01

**Authors:** Alin Cristian Nicolae Vintila, Mircea Vinatoru, Ana-Maria Galan, Alexandru Vlaicu, Mihaela Ciltea-Udrescu, Anca Paulenco, Adina Ionuta Gavrila, Ioan Calinescu

**Affiliations:** 1 National Institute for Research & Development in Chemistry and Petrochemistry—ICECHIM, 202 Spl. Independentei, 060021 Bucharest, Romania; 2 Faculty of Chemical Engineering and Biotechnologies, University Politehnica of Bucharest, 1-7 Polizu Street, 011061 Bucharest, Romania

**Keywords:** microalgae, ultrasound, extraction, PUFA

## Abstract

The influence of ultrasound irradiation on the algal biomass productivity as well as its oil content and fatty acids profile, grown in a modified Zarrouk medium, i.e., deproteinized whey waste solution, was investigated. The algal samples (*Nannochloris* sp. 424-1 microalgae) were grown for 7 days in a thermostated incubator at 28 °C, shaken under continuous light. During this period, the algal biomass was subjected to induced stress by ultrasonic irradiation at different powers and sonication time. The obtained results demonstrate that ultrasound stressing of algae biomass has a positive effect on both the quantity of biomass and the oil obtained, also causing a shift in fatty acid composition by increasing the proportion of C16 and C18 polyunsaturated fatty acids. A low dosage level of exposure to the ultrasound led to algal biomass increase as well as lipid accumulation. For both types of irradiation modes which were investigated, daily and only initial irradiation, the beneficial effect of the ultrasound decreases as the exposure time increases and the excessive sonication becomes detrimental to microalgae growth.

## 1. Introduction

Microalgae are of great interest because of their potential to offer a higher production of biomass that does not compete with agricultural crops planned for human consumption [1]. Moreover, the use of sunlight is more efficient in producing useful compounds in algae via photosynthesis compared with land species. Algae represent an untapped source of high value-added products such as proteins, lipids and carbohydrates, which in turn can serve as feedstock for a wide array of industries. Microalgae biomass can be also used as a food supplement for the human diet or in animal husbandry [2,3,4].

A consensus regarding the definition of stress in the context of biochemical processes is hard to reach; even the classification of stress factors is under debate. Some researchers account for the nature of the stress factor (physical, nutritional, biochemical, etc.), while others refer to the outcome of the applied stress. A broad definition can be considered as being the response of any biological system as a result of changes which occur in their environmental conditions [5]. The versatility of microalgae species allows them to adapt to the variations in their growth medium and modifications of their cultivation conditions. These growth medium variations could be exploited by growing algae strains under stressing factors to favor the accumulation of specific biochemicals [5].

Altering the available nutrients is one of the most common ways to direct growing algae towards production and accumulation of useful biocomponents, for example by feeding with different carbon sources such as glucose, glycerol, acetic acid or lactose from the dairy industry wastewaters [6,7]. The diversity in potential of carbon sources for feeding the microalgae abovementioned will lead them to undergo different metabolic pathways; therefore, there are changes in the compositions of the primary metabolites [2].

The use of ultrasounds for algae treatment has widely been directed towards enhancing extraction processes due to the ability to improve cell permeability and transportation across the membrane of the substrate or to rupture the cells, releasing their compounds i.e., lipids [8,9]. A less common method was the use of ultrasound for microalgae growth stimulation to increase its biomass and lipid production. Ren et al. [10] observed that an applied ultrasound during the growth phase of *Scenedesmus* sp. had a reduced effect on the survivability of microalgae cells, and instead led to an increase in biomass and lipid production. Thus, a maximum value of 2.78 g/L biomass productivity and 28.5% lipid content was achieved when the ultrasound of 20 Hz frequency and 20 W power was used for a 2 s interval [10].

Lipid accumulation has been proven to be induced by a combination of multiple factors, more commonly dual stress in the form of nitrogen limitation and a different environmental stress factor, such as light [11]. In order to overcome the opposite trends between biomass accumulation and lipid content, a two-stage cultivation strategy was proposed, consisting of initial optimal conditions for biomass accumulation, followed by lipid promoting cultivation in the presence of stress factors [12]. Wei et al. successfully applied this two-stage strategy for the microalgae strain *Tetradesmus obliquus*, resulting in an increase of 32.1% for biomass production and 44.5% for lipid content, when nitrogen limitation and low-frequency ultrasonic treatment was applied [13].

The aim of this study is to investigate the effect of ultrasonic treatment, with two selected ultrasonic powers at 26 kHz, throughout the growth period of the *Nannochloris* sp. 424-1 microalgae strain, cultivated in whey, which is considered a waste in the dairy industry upon the production of algal oil.

## 2. Materials and Methods

The *Nannochloris* sp. 424-1 microalgae strain was used in this study, which is available at INCDCP – ICECHIM as a patented microalgae strain [14], deposited under the code CCAP 251/10 at the Culture Collection of Algae and Protozoa at the Scottish Marine Institute, Aryl, UK.

The growth of this microalgae was carried out in a modified medium prepared from solutions of deproteinized whey by dilution in the optimal Zarrouk growth medium, respectively: Solution 1–13.61 g NaHCO_3_, 4.03 g Na_2_CO_3_, 0.5 g K_2_HPO_4_ per 0.5 L and Solution 2–2.5 g NaNO_3_, 1 g K_2_SO_4_, 1 g NaCl, 0.2 g MgSO_4_·7H_2_O, 0.04 g CaCl_2_, 0.01 g FeSO_4_·7H_2_O, 0.08 g Na_2_EDTA per 0.5 L. After sterilization at 121 °C for 15 min, the two solutions were mixed, and 1 mL/L of microelements solution was added. The composition of microelements solution per liter is as follows: 2.86 g H_3_BO_3_, 2.03 g MnSO_4_·4H_2_O, 0.222 g ZnSO_4_·7H_2_O, 0.018 g MoO_3_, 0.079 g CuSO_4_·5H_2_O, 0.494 g Co(NO_3_)_2_·6H_2_O.

**Figure 1:**
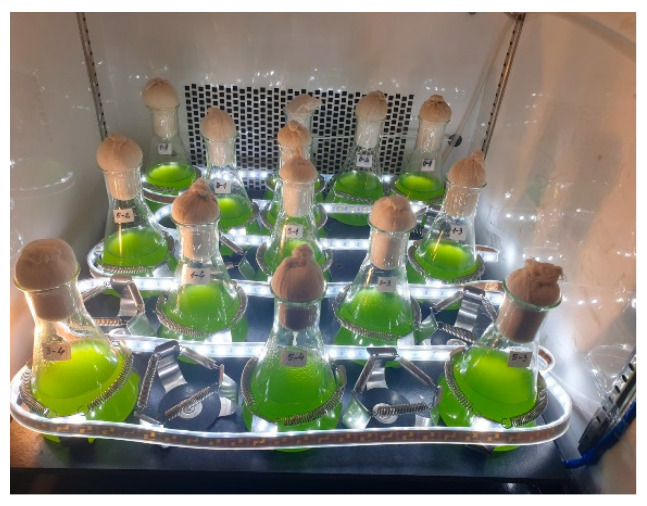
Experimental setup.

Locally sourced cheese whey of bovine origin was used for sample preparation. The initial whey treatment step consisted of deproteinizing through boiling the solution, followed by cooling and precipitation. The separated proteins were removed by centrifugation and filtration. The initial lactose concentration in the deproteinized whey solution (45.45 g/L) was determined using a spectrophotometric method based on the dinitrosalicylic acid (DNSA) assay for reducing the sugars, published by Miller [15]. The characteristics of the raw cheese whey after deproteinizing are as follows: chemical oxygen demand (COD) 58,000 mg/L, pH 6.2, salinity 3%, total phosphorus (TP) 1435 mg/L and total nitrogen (TN) 282 mg/L. Experiments were carried out in a thermostated Innova 42R (New Brunswick, Eppendorf, Germany) incubator at 28 °C for 7 days, under light (240 µmol photons m^−^^2^·s^−^^1^) and continuously shaken, as seen in Figure 1. All samples were inoculated in Erlenmeyer flasks with a total volume of 200 mL, consisting of 20 mL inoculum, 169 mL Zarrouk medium and 11 mL deproteinized whey, so as to ensure an initial lactose concentration of 2.5 g/L. All experiments were performed in duplicate.

To induce a growth stress to this microalgae strain, two ultrasonic irradiation methods were used:

(a) Initial US irradiation, meaning that the algal growing mixture was irradiated just once with the specific power (each sample 1, 3 and 5 min time for 1 W and 6, 18 and 30 s for 10 W).
(b) Daily US irradiation, meaning that during the experiments, the algal biomass was daily irradiated using similar times for each US power.

The effect of ultrasound was observed by comparing exposed with control samples. The control samples were grown using the same medium and growth conditions, but in the absence of an ultrasound.

The ultrasonic equipment used was a Hielscher UP200St-G of 26 kHz capable of measuring and reporting the power and energy introduced into the system, with a maximum power up to 200 W. This equipment has a glass sonotrode (Duran) with a diameter of 26 mm. The exposure times were adjusted between the two powers (as pointed out above) to ensure comparable total ultrasonic energy (in J/mL) supplied to the samples.

Table 1 shows the energy values transmitted to the algal sample for different powers and times. The power densities (W/mL) used are very low to not destroy the algae. The energy values are those indicated by the US device. It can be observed that the chosen irradiation time was so that the energy values were very close for both employed ultrasonic powers.

The ultrasonic power and the irradiation time give almost the same energy for each type of experiment. Thus, 1 W ultrasonic power and 60 s irradiation time provide almost the same energy as for 10 W, in only 6 s. However, it is impossible to totally deny the collapsing cavitation bubbles occurring at 10 W. This is visible in the results obtained and discussed below.

Microalgae harvesting was carried out via centrifugation using a Rotina 420R Hettich centrifuge, at 8500 rpm for 20 min. The biomass was washed with distilled water to remove salts which may have been embedded by the cells. Lyophilization was used as the drying method for the harvested biomass.

Biomass productivity (g/L) was obtained by taking the weight of each dried sample and dividing it by the respective volume of microalgal suspension. After grinding the biomass using a mortar and pestle, samples were subjected to lipid extraction using a modified Folch method [16]. This involved adding the ultrasound in the extraction stage with chloroform:methanol [2:1 (*v*/*v*)], sonicating each sample for 20 min, and using an ELMA TRANSSONIC T420 ultrasonic bath with a solvent (v) to biomass (w) ratio of 4:1 [17]. The process was repeated 3 times for each sample and the liquid extracts were reunited before drying and weighting the lipid fraction. Fatty acid profiles were obtained by GC—MS analysis of transesterified samples, after reactions with methanolic solutions of 4% KOH for 120 min and 20% BF_3_ for 90 min at reflux, using a ratio of 1:20:27 (*w*/*v*/*v*) [17].

For statistical analysis, triplicate measurements (*n* = 3) were carried out with the data obtained being expressed as mean value ± standard deviation (SD) for each triplicate. One-way analysis of variance technique (ANOVA) was used for all results. The data were also analyzed by performing multiple comparison post hoc t-tests, used to establish the significance of the statistical differences between the measurements’ averages of two or more independent groups. The statistically significant differences were considered as those at a minimal level of significance of *p* < 0.05.

## 3. Results and Discussion

**Figure 2:**
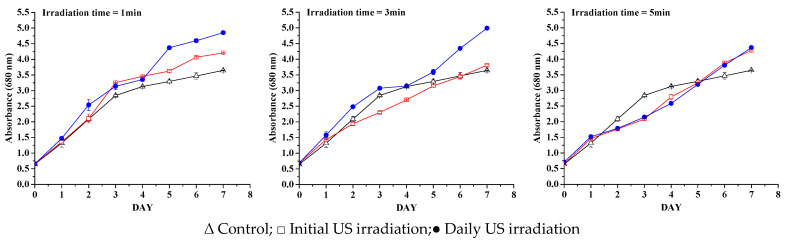
Growth curves for samples exposed at 1 W ultrasonic power.

**Figure 3:**
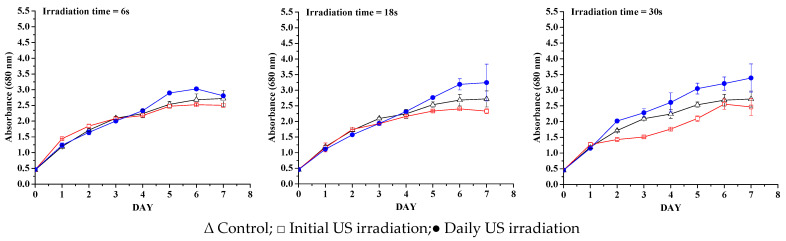
Growth curves for samples exposed at 10 W ultrasonic power.

The growth of *Nannochloris* sp. 424-1 was monitored by daily sampling, which was analyzed spectrophotometrically at 680 nm. Spectrophotometric analysis of the microalgae sample, which was used as inoculum in the 530–800 nm range, resulted in a maximum absorption peak at 680 nm, with a full width at half maximum (FWHM) of 68.5. This was based on the correlation between chlorophyll and cell growth, which has been widely used in the past for green microalgae [18,19]. Multiple methods have been proposed in the existing literature for correlating microalgae growth with absorbance and optical density, but the accuracy of spectrophotometric methods is often insufficient, which is why biomass productivity was used as the main indicator of the effect of ultrasound on cell growth; while, the spectrophotometric curves were mainly useful to highlight whether the exposure to an ultrasound led to a delay in metabolic activity [20]. The growth curves created based on analyzed data are presented in Figure 2 and Figure 3, for 1 and 10 W, respectively. The growth curves obtained by measuring the absorbance of microalgae samples are a good indicator of the efficiency of the metabolic activity of cells during the growth process, given the excellent correlation between absorbance/optical density and dried algae biomass [21].

At 3 min daily exposure and 1 W ultrasonic power (Figure 2), a positive effect on the growth of microalgae was observed (not optimized), compared with the microalgae only initially exposed to ultrasound. Regardless of the exposure times and irradiation mode, in the case of a lower ultrasonic power, all samples had a higher productivity over the control samples. When the ultrasonic power was increased to 1.9 W/cm^2^ intensity (10 W US power), with the exposure time lowered to match the ultrasonic power exposure for 1 W power (close to time/total energy for each US power used, as shown in the Table 1), only the samples that were irradiated daily showed a positive effect on biomass productivity. This can be translated by the fact that the stress memory induced by initial irradiation is not enough, except for the low power and longer irradiation time, of which both the daily and only initial sonication end up resulting in the same absorbance value after one week. The algal growth in the case of 10 W is lower compared with 1 W ultrasonic power, indicating that, even if the ultrasonic power intensity in the case of 10 W is less than the accepted cavitation threshold limit [22,23,24], damaging cavitation collapse is not totally avoided and so a part of the algae were destroyed by ultrasonic irradiation. This is visible in the graphs (Figure 3). To determine whether the results were statistically significant, a t-test was conducted. The results of the t-test were statistically significant, with a *p*-value < 0.05. This means that there is a significant difference between the stress levels of the two groups. The one-way ANOVA analysis along with a multiple comparison post hoc test (*n* = 8) showed that the irradiation with an ultrasonic power of 1 W (Figure 2), in both the initial irradiation and the daily irradiation, has a significant effect (*p*-values < 0.05) on the growth of microalgae. When the samples are irradiated with an ultrasonic power of 10 W (Figure 3), it is observed that only daily irradiation has a significant effect on the growth of microalgae (*p*-values < 0.05).

**Figure 4:**
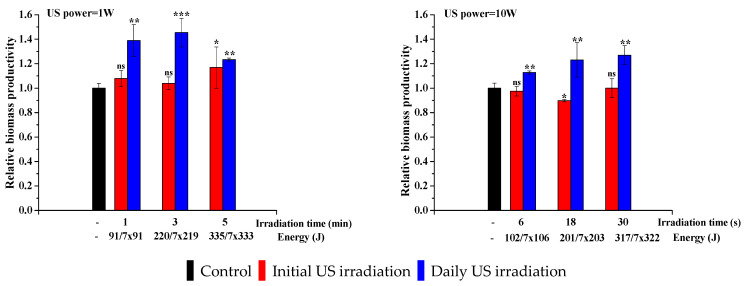
Effect of ultrasound on biomass productivity (normalized to control sample, whose value is 0.777 g/L). Data were analyzed using one-way ANOVA (*p* < 0.05) and multiple comparison post hoc t-tests (*n* = 6). Asterisks indicate the significant difference between each group, compared with the control values (* = *p* < 0.05, ** = *p* < 0.01, *** = *p* < 0.001, and ns = no significant difference).

When this type of stress is used it is important to monitor all parameters: biomass productivity and the amount of oil produced at the end of 7 days of cultivation. Figure 4 shows that, again, a lower ultrasonic power proves to be more effective than the control. A maximum increase in biomass productivity of 45% was obtained for 3 min of daily exposure to an ultrasonic power of 1 W. When using 10 W ultrasonic power, initial irradiation was insufficient for a beneficial effect of ultrasound on microalgae growth to be noticeable. However, daily irradiation, even in such intense stress conditions, led to a positive increase of 27% over the control sample after 30 s of ultrasonic irradiation. This shows that the ultrasonic stress is effective, allowing the algae to adapt but only at lower acoustic power. Longer periods of sonication time or high ultrasonic powers are too much for the microalgae to adapt. The ANOVA analysis along with multiple comparison post hoc tests (*n* = 6) demonstrated that in the case of irradiation with an ultrasound power of 1 W (Figure 4), the results are significant (*p*-value < 0.05) both at the initial irradiation and with daily irradiation, as well as in comparisons with the control. Upon irradiation with an ultrasound power of 10 W, the multiple comparison post hoc tests (*n* = 6) demonstrated that only daily irradiation leads to a significant increase in biomass productivity (*p*-value < 0.05).

**Figure 5:**
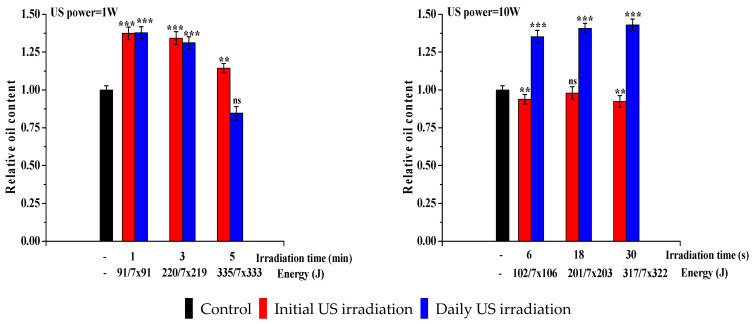
Effect of ultrasound on oil content (normalized to control sample, whose value is 0.037 g oil/g dry biomass). Data were analyzed using one-way ANOVA (*p* < 0.05) and multiple comparison post hoc t-tests (*n* = 6). Asterisks indicate the significant difference between each group, compared with the control values (** = *p* < 0.01, *** = *p* < 0.001, and ns = no significant difference).

The most important parameter is the algal oil content, being the reason for ultrasonically stressing the algae. Figure 5 shows the oil content for each sample, in both types of irradiation modes and for both ultrasonic powers, expressed as grams oil/grams biomass obtained after full processing of microalgal biomass throughout oil extraction and its subsequent transesterification. It can be observed that when a lower ultrasonic power is used, a limited exposure period (1 min) is beneficial towards oil production and accumulation of the microalgal biomass, with an up to 38% increase for both types of ultrasonic irradiation (initial and daily). When the intensity of the ultrasonic treatment was increased to 10 W and the exposure time was reduced to seconds, the use of ultrasound only as an initial stimulus was insufficient, and oil content levels stayed close to or slightly below the control sample. Daily ultrasonic irradiation at 10 W leads to higher oil contents compared with the control sample; however, the intensity of the treatment mode does not allow for values as high as those obtained when 1 W of ultrasonic power is applied to the microalgae samples. The ANOVA analysis along with multiple comparison post hoc tests (*n* = 6) demonstrated that, in both cases (Figure 5), the results are significant (*p*-value < 0.05) both for the initial irradiation and for the daily irradiation compared with control samples. Regarding irradiation with an ultrasound power of 10 W, the multiple comparison post hoc tests (*n* = 6) demonstrated that only daily irradiation leads to a significant increase in oil content (*p*-value < 0.05).

**Figure 6:**
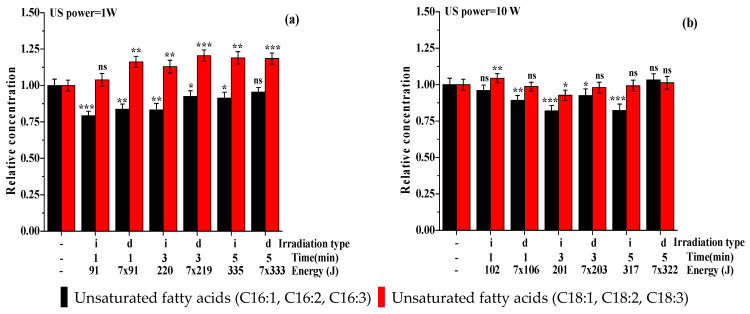
Effect of ultrasound (a = 1 W, b = 10 W) on fatty acids composition (normalized to control sample, whose value is 24.28% for unsaturated fatty acids with 16 C atoms and 47.72% for unsaturated fatty acids with 18 C atoms). Data were analyzed using one-way ANOVA (*p* < 0.05) and multiple comparison post hoc *t*-tests (*n* = 6). Asterisks indicate the significant difference between each group, compared with the control values (* = *p* < 0.05, ** = *p* < 0.01, *** = *p* < 0.001, and ns = no significant difference).

It seems, however, that ultrasonic induced stress, low power, has an effect not only on the algal biomass but also on the oil content and fatty acid’s structure. Thus, a shift in the fatty acid profile can be observed, especially for the case in which 1 W of ultrasonic power is applied, with a decrease in C16 unsaturated fatty acids and an increase in C18 unsaturated fatty acids for both types of irradiation modes (Figure 6a). When an intensity of 10 W ultrasonic power is applied, both categories of unsaturated acids are reduced (Figure 6b). A possible explanation for this effect could be that, as a response to stress, the amount of protective oil (rich in C18 fatty acids) is induced in algae. In order to determine if the fatty acid profile differed compared with a normalized control sample, a *t*-test was conducted. The results of the *t*-test were statistically significant, with a *p*-value < 0.05. This means that there is a significant difference between the stress levels of the two groups. The ANOVA analysis along with multiple comparison post hoc tests (*n* = 6) demonstrated that, in both cases (Figure 6), the results are significant (*p*-value < 0.05) both for the initial and daily irradiation compared with the control samples. By irradiation with an ultrasound power of 1 W, the multiple comparison post hoc tests (*n* = 6) demonstrated that the C18 fatty acids relative concentration increased significantly (*p*-value < 0.05). By irradiation with an ultrasound power of 10 W, the multiple comparison post hoc tests (*n* = 6) demonstrated that only daily irradiation does not significantly affect the fatty acid content (Figure 6b).

There have been quite a few studies conducted to highlight the influence of the US on algal growth [25]. Our research illustrated that an ultrasonic glass probe with a large diameter (26 mm) was used to stress the algal biomass. However, avoiding destruction of it is possible if the power intensity does not exceed the cavitation threshold within the volume of algal biomass. Thus, at the power of 1 W, the power intensity was only 0.19 W/cm^2^, while at 10 W it was 1.9 W/cm^2^. In addition, the specific power density input, calculated as W/mL, was very small, being between 0.05 for 10 W and 0.005 for 1 W power.

The higher economic value of unsaturated fatty acids with 18 carbon atoms compared to unsaturated acids with 16 carbon atoms points to the fact that the effect of controlled ultrasonic irradiation of this strain of algae has a valuable potential.

The literature data presented in Table 2 show the effect of an ultrasound applied during growth on the biomass productivity and lipid content of another species of microalgae, *Scenedesmus* sp., thus highlighting the positive influence of ultrasound. The results of this are in line with those obtained in this work for the species *Nannochloris* sp.

## 4. Conclusions

Results obtained for *Nannochloris* sp. highlight the potential of ultrasound as a beneficial and valuable treatment method for enhancing microalgae growth. Both biomass productivity and lipid content have been monitored throughout these experiments and have been shown to benefit from exposure to ultrasound stress. Two ultrasonic powers have been used in this study, 1 W and 10 W, with exposure times adjusted between the two treatment intensities to ensure similar ultrasonic energies are supplied to the system. A smaller intensity dosage of ultrasound led to an increase in biomass, lipid accumulation and a beneficial oil profile over control samples. In both types of irradiation modes, daily exposure and initial irradiation, the beneficial effect becomes less significant, with increases in the exposure time, as the damage generated by excessive sonication becomes detrimental to microalgae growth. When increasing the intensity of the ultrasonic treatment, only daily exposure to the ultrasound allows for a positive effect to manifest. Yet, the increase in either biomass or oil is smaller than that obtained for the lower ultrasonic power. This shows that when an ultrasound is chosen as a treatment option for microalgae growth or lipid accumulation, the ultrasonic dose shouldn’t be the only parameter taken into account. Both ultrasonic power and exposure time affect microalgae cells, and ignoring them leads to an inefficient or destructive treatment.

## Figures and Tables

**Table 1:** The energy transferred to the sample as a function of power and time.

US Power, W	Specific Power W/mL	Power Intensity * W/cm^2^	Treatment time/Energy (s/J)		
1	0.005	0.19	60/91	180/219	300/333
10	0.05	1.9	6/106	18/203	30/320

* at the tip of sonotrode.

**Table 2:** The influence of ultrasound on the biomass productivity and lipid content.

Strain	Ultrasonic Treatment	Biomass Productivity (g/L)		Lipid Content (g/L)		Reference
		Control	Ultrasound	Control	Ultrasound	
*Scenedesmus* sp.	20 W 2 s on/2 s off 4 min	1.91	2.68	0.76	1.31	[8]
*Scenedesmus* sp. Z-4	20 W 20 Hz 2 s	2.19	2.78	0.58	0.79	[10]
*Scenedesmus* sp.	20 W 18 Hz 10 min	0.69	1.56	0.166	0.24	[26]

## Data Availability

The data presented in this study are available on request from the corresponding author.
